# The Acceptability of Technology-Based Physical Activity Interventions in Postbariatric Surgery Women: Insights From Qualitative Analysis Using the Unified Theory of Acceptance and Use of Technology 2 Model

**DOI:** 10.2196/42178

**Published:** 2023-01-23

**Authors:** Pierre Thérouanne, Meggy Hayotte, Florent Halgand, Fabienne d'Arripe-Longueville

**Affiliations:** 1 Université Côte d'Azur, LAPCOS Nice France; 2 Université Côte d'Azur, LAMHESS Nice France

**Keywords:** acceptability, health technology, physical activity, obesity, UTAUT2

## Abstract

**Background:**

Bariatric surgery offers an opportunity for physical activity (PA) promotion due to patients’ increased ability to engage in PA. Technology-based PA interventions are promising tools for promoting PA to support patients in this key period. The Unified Theory of Acceptance and Use of Technology 2 (UTAUT2) model is a recognized theoretical model for examining technology acceptability. Although a previous study reported that 92% of women with obesity have high acceptability of at least one technology-based PA intervention, little is known about the factors that lead to different levels of acceptability between technologies and therefore the reasons for choosing a preferred intervention.

**Objective:**

The purpose of this study was to (1) characterize the acceptability of 3 technology-based PA interventions (ie, telehealth, active video game, mobile app) in the context of bariatric surgery, and (2) explore patients’ preference motives. This study, using a qualitative design, examined the suitability of the UTAUT2 model in this specific context.

**Methods:**

Participants (n=26) read written French descriptions of the technology-based PA interventions with illustrations and chose their preferred intervention. Semidirective interviews were conducted to explore the reasons for their choice of the preferred intervention, notably using the UTAUT2 framework. Data were analyzed based on inductive and deductive approaches.

**Results:**

All participants who preferred a technology-based PA intervention (ie, active video game, n=10; mobile app, n=10; telehealth, n=6) expressed a behavioral intention to use it. In addition, some of them expressed a high behavioral intention to use another technology (ie, active video game, n=4; mobile app, n=1; telehealth, n=7). All the constructs of the UTAUT2 emerged during the qualitative interviews and were specified through subcategories. Additional constructs also emerged, especially other motivational factors.

**Conclusions:**

This study showed that, in the context of technology-based PA interventions for postbariatric patients, the UTAUT2 is suitable, although additional motivational factors (which were not considered by the UTAUT2 model) should be considered.

## Introduction

Technology-based physical activity (PA) interventions have been increasingly investigated in recent years to promote PA for vulnerable populations. These interventions have been used effectively to promote PA in the context of obesity care [1-4]. A recent meta-analysis confirmed that they were able to increase moderate-to-vigorous PA for women with obesity by approximately 25 minutes per week [5]. We also note an emerging interest in technology-based PA interventions in the context of bariatric surgery [6]. Bariatric surgery induces major weight loss that is perceived by patients as an increase in their ability to engage in PA [7]. In addition, PA is a favorable factor for long-term weight loss maintenance [8]. However, many postbariatric patients do not increase their PA and some even decrease it [9]. Women, especially young women, represent a higher proportion of bariatric surgery patients than men [10] and seem to be more prone to physical inactivity and sedentary behavior [11,12]. Thus, young women after bariatric surgery offer a good example of a chronic disease population in a key period to induce behavior change.

To this end, 3 categories of technology-based PA interventions can be recommended to patients: mobile technology (eg, mobile apps, wearable devices), game-based interventions (eg, active video games, exergames, serious games, augmented and virtual reality games), and computer- and internet-based interventions (eg, telehealth, email, websites, social media) [13-16]. Some technology-based PA interventions are more preferred (ie, more accepted) than others [17]. However, little is known about the preference motives of postbariatric surgery patients. Thus, characterizing the acceptability of technology-based PA interventions in this context would encourage the individualization of the recommendations for a given intervention based on the patient profile. Doing so would also provide engineers with information on patients’ preference motives that could guide them in adapting or developing new adapted technology–based PA interventions tailored to postbariatric surgery patients.

The reasons why some tools are chosen, accepted, and used more than others can be explained by models of acceptability [18]. Among the models, the Unified Theory of Acceptance and Use of Technology 2 (UTAUT2) [19] is today the most comprehensive, parsimonious, and powerful predictive model of the behavioral intention to use technology [20,21]. The UTAUT2 model is an extension of the UTAUT to a consumer context [19]. The model assumes that performance expectancy, effort expectancy, social influence, and facilitating conditions are key constructs that influence behavioral intention to use a technology or technology use [22]. The UTAUT2 incorporates 3 additional constructs, namely, hedonic motivation, price value, and habit [19]. The UTAUT2 constructs are defined as follows: (1) *performance expectancy* refers to “the degree to which using a technology will provide benefits to consumers in performing certain activities,” (2) *effort expectancy* refers to “the degree of ease associated with consumers’ use of technology,” (3) *social influence* refers to “the extent to which consumers perceive that important others (eg, family and friends) believe they should use a particular technology,” (4) *facilitating conditions* refers to “consumers’ perceptions of the resources and support available to perform a behavior,” (5) *hedonic motivation* refers to “the fun or pleasure derived from using a technology,” (6) *price value* refers to “consumers’ cognitive trade-off between the perceived benefits of the technology and the monetary cost of using it,” and (7) *habit* refers to “the extent to which an individual believes the behavior to be automatic” [19].

This model has been adapted into French in the context of eHealth [23]. Moreover, studies have recently investigated the relevance of the UTAUT2 model in certain chronic diseases. For example, in the case of diabetes, all UTAUT2 constructs were found to be relevant and 2 additional constructs, trust and perceived disease threat, also emerged as predictors of mHealth acceptability [24]. Several studies have also extended the UTAUT2 model to a variety of contexts that can be grouped into 6 categories: (1) different types of users, (2) different types of organization, (3) different types of technology, (4) different task types, (5) different times, and (6) different locations [25]. However, this model has rarely been used in the specific context of PA interventions, and even less so after bariatric surgery, which is a good example of a critical period for behavior change.

In the context of obesity care, including care for bariatric surgery patients, a latent profile analysis identified 2 acceptability profiles: (1) a high acceptability profile (ie, n=230 for telehealth, n=235 for active video game, and n=257 for mobile app), and (2) a low acceptability profile (ie, n=82 for telehealth, n=77 for active video game, and n=55 for mobile app) [17]. This study also demonstrated that these acceptability profiles were related to motivational factors (which were not considered by the UTAUT2 model). Although 92% of the women with obesity were in a high acceptability profile for at least one of the three technology-based PA interventions, this study did not account for the factors that led to different levels of acceptability between technologies. Therefore, it provided no information about the specificities of the different UTAUT2 constructs in the context of technology-based PA interventions for postbariatric surgery patients (ie, the items measuring the UTAUT2 constructs are generic and therefore not specifically tailored to this context), nor about their preference motives.

This study aimed to (1) characterize the acceptability of 3 technology-based PA interventions (ie, telehealth, active video game, mobile app) in the context of bariatric surgery; and (2) explore patients’ preference motives. Using a qualitative design, the study examined the suitability of the UTAUT2 model in this specific context.

## Methods

### Procedure

Individuals were invited to participate in this study in the waiting rooms for their routine postbariatric surgical care appointments in the South of France after participation in a previous quantitative study [17]. Eligible participants had read the written French descriptions of 3 technology-based PA interventions with illustrations in a counterbalanced order following a Latin-square design: active video game, mobile app, and telehealth (Multimedia Appendix 1). After reading the descriptions, they classified the technology-based PA interventions according to their preferences and were asked if they would be willing to participate in a follow-up interview to explore in-depth their preference motives.

The interview was conducted on the same day as completion of the questionnaires of the previous study or at the next follow-up appointment (with a maximum delay of 6 months), or by phone, with a mean delay of 51.0 (SD 68.3) days. This delay was chosen to limit patient burden and seemed reasonable as no technology-based PA interventions were offered to the patients during this period. These 1-on-1 interviews were conducted in a specialized obesity center and organized in conjunction with outpatient visits or by phone by FH between June and December 2019. The descriptions of the interventions were presented again at the beginning of the interviews, which lasted a mean 17.3 (SD 5.2) minutes. Participants were asked to provide demographic data including (1) year of birth, (2) sex, (3) marital status, (4) educational level, and (5) self-reported height (m) and weight (kg) used for calculating the BMI (kg/m^2^). Four researchers analyzed the data; 2 researchers were specialized in psychology and ergonomic sciences (FH and PT) and 2 were from the fields of exercise psychology and social psychology (MH and FA-L). Interviews were audio-recorded and transcribed verbatim. Interviewing ended when theoretical saturation was reached at the general level for all technology-based PA interventions combined [26]. Theoretical saturation is a guiding principle classically used to assess sample adequacy in qualitative research. Recently, a systematic review of empirical tests showed that 9-17 interviews reach saturation for a homogenous study population with narrowly defined objectives [27].

### Ethics Approval

The study was conducted in accordance with the Helsinki principles and was recorded by the Data Protection Officer of Université Côte d’Azur (records of processing activities number UCA-E009). All participants gave their electronic informed consent before participation.

### Participants

#### Inclusion Criteria

Inclusion criteria were the following: (1) women residing in France, (2) between 18 and 40 years of age, (3) having undergone bariatric surgery at least two months earlier, (4) with care received in the south of France, (5) without PA limitation, and (6) speaking French fluently. We focused on women because they undergo bariatric surgery more often than men and make up 82% of those undergoing this surgery in France [28]. Moreover, women undergo bariatric surgery at a younger age [29] and are more prone to physical inactivity and sedentary behaviors [11] than men. We restricted the inclusion criteria to young women to ensure sample homogeneity and to avoid confounding by UTAUT2 moderators such as age and sex.

We had performed an earlier quantitative study with patients with obesity about the acceptability of 3 technology-based PA interventions [17]. Among the 133 eligible participants, (1) 54.9% (n=73) preferred mobile app as their first choice (other first choices: n=42 preferred active video game and n=18 preferred telehealth); (2) 46.6% (n=62) preferred active video game as their second choice (other second choices: n=41 preferred mobile app and n=30 preferred telehealth); and (3) 63.9% (n=85) preferred telehealth as their third choice (other third choices: n=29 preferred active video game and n=19 preferred mobile app). As many as 26 of these women (preference choice: active video game, n=10; mobile app, n=9; and telehealth, n=7) volunteered to participate in this study. This subsample was not representative of the previous 133 participants in terms of preferences (ie, as a first choice, n=73, 54.9%, chose mobile app; n=42, 31.5%, chose active video game, and n=18, 13.5%, chose telehealth). As the objective of the study was to explore patient preference motives, we preferentially conducted the interviews with a view to balancing the number of participants preferring each of the technology-based PA interventions until theoretical saturation was achieved.

#### Interview Guide

The interview guide was mainly based on the constructs of the UTAUT2 model [19]. A pilot interview enabled us to reformulate some of the questions and focus the interview on the preferred technology. The final guide comprised 4 parts: (1) presentation of the descriptions of the technology-based PA interventions and confirmation of the ranked preferences, (2) exploration of the reasons for the ranking, (3) application of the UTAUT2 dimensions to the preferred technology and comparison with the other interventions, and (4) exploration of other factors that could influence acceptability (Multimedia Appendix 2).

### Qualitative Analysis

Qualitative analysis was conducted in several steps according to the qualitative research guidelines [26,30-32]. In the first step, PT and FH determined the segmentation procedure based on Strijbos et al [33] independently of the coding categories of our study. Data were segmented based on punctuation and subdivided when a segment included several units of meaning; conditional relations constituted 1 segment, and sentences left pending and speech tics were excluded. In the second step, PT and FH read the units of meaning several times to become familiar with the data. They coded the units deductively into the main dimensions of the UTAUT2 model [19]: (1) performance expectancy, (2) effort expectancy, (3) social influence, (4) facilitating conditions, (5) hedonic motivation, (6) price value, (7) habit, and (8) behavioral intentions. They then determined the subcategories of the UTAUT2 dimensions inductively. Units of meaning that were not relevant for the UTAUT2 dimensions were organized into emergent new categories and subcategories. The 2 researchers independently coded 30% of the data (ie, 8 interviews out of 26) and obtained 94.02% agreement (ie, 1132 units of meaning were coded identically out of 1204 units). They then shared their coding and discussed any diverging results until agreement was reached. The rest of the data coding was then shared out between PT and FH. In the fourth step, FA-L and MH reviewed the categories and codes as “disinterested peers” to strengthen the qualitative research validity [34]. Category labels were refined with the agreement of all researchers. As the UTAUT2 dimensions are defined as degrees, PT and MH specified independently for each participant whether the category cited was perceived positively, negatively, or neutrally. The authors obtained 93.74% agreement (ie, 494 codes were perceived identically out of 527 codes) and resolved disagreements by consensus. They then counted the number of participants who reported each category for each technology-based PA intervention. As a final step, relevant and short extract examples were identified and selected with the agreement of all researchers.

## Results

### Demographic Statistics

A total of 26 women volunteers aged 18-40 years who had undergone bariatric surgery participated in this study. Demographic statistics are listed in Table 1.

**Table 1:** Sociodemographic characteristics (n=26)

Characteristics		Values
Age (years), mean (SD)		32.9 (5.5)
Body mass index (kg/m^2^), mean (SD)		30.1 (6.5)
**Education (years), n (%)**		
	<12	10 (38.5)
	12	9 (34.6)
	14-15	7 (26.9)
	≥17	0 (0)
**Professional status, n (%)**		
	Employed	20 (76.9)
	Unemployed	5 (19.2)
	Student	1 (3.8)
**Marital status, n (%)**		
	Single or never married	10 (38.5)
	Married or in a civil union	12 (46.2)
	Divorced or widowed	4 (15.4)

### Qualitative Analysis

#### Overview

Units of meaning for each technology-based PA intervention were coded deductively into the main dimensions of the UTAUT2 model. Then, subcategories of the UTAUT2 dimensions were determined inductively. Codes that were not relevant for the UTAUT2 dimensions were organized into emergent new categories and subcategories (Table 2). The way each participant perceived the different acceptability categories and subcategories is reported in Multimedia Appendix 3. These perceptions are also summarized in Table 2.

**Table 2:** Prevalence and valence^a^ of acceptability categories and subcategories cited by the participants (n=26) for each technology-based physical activity intervention^b^.

Categories and subcategories					Active video game			Mobile app			Telehealth
			n (%); valence^a^			n (%); valence			n (%); valence		
**UTAUT2 constructs**											
	**Performance expectancy**			*19 (73.1)*			*19 (73.1)*			*17 (65.4)*	
		Adequacy of PA^c^	8 (30.8); 5 (–), 1 (+), 2 (±)			8 (30.8); 2 (–), 4 (+), 2 (±)			12 (46.2); 12 (+)		
		Engagement and sustainability of PA	16 (61.5); 1 (–), 14 (+), 1 (±)			12 (46.2); 2 (–), 9 (+), 1 (±)			14 (53.8); 1 (–), 13 (+)		
		PA management support	3 (11.5); 1 (–), 2 (+)			13 (50.0); 10 (+), 3 (±)			4 (15.4); 4 (+)		
	**Effort expectancy**			*11 (42.3)*			*14 (53.8)*			*5 (19.2)*	
		Effort required by PA	6 (23.1); 3 (–), 2 (+), 1 (±)			4 (15.4); 3 (+), 1 (±)			2 (7.7); 2 (+)		
		Effort required by the technology	8 (30.8); 2 (–), 4 (+), 2 (±)			14 (53.8); 5 (–), 9 (+)			4 (15.4); 4 (+)		
	**Social influence**			*12 (46.2)*			*11 (42.3)*			*5 (19.2)*	
		Others’ perceptions on the technology-based PA interventions	8 (30.8); 1 (–), 5 (+), 2 (±)			10 (38.5); 8 (+), 2 (±)			4 (15.4); 2 (+), 2 (±)		
		Others’ uses of the technology-based PA interventions	6 (23.1); 1 (–), 4 (+), 1 (±)			3 (11.5); 3 (+)			1 (3.8); 1 (+)		
	**Facilitating conditions**			*22 (84.6)*			*24 (92.3)*			*20 (76.9)*	
		Anytime and anywhere usage	17 (65.4); 7 (–), 6 (+), 4 (±)			22 (84.6); 2 (–), 18 (+), 2 (±)			18 (69.2); 11 (–), 5 (+), 2 (±)		
		Available material resources	11 (42.3); 2 (–), 5 (+), 4 (±)			11 (42.3); 10 (+), 1 (±)			9 (34.6); 3 (–), 5 (+), 1 (±)		
		Technological knowledge	3 (11.5); 1 (–), 2 (+)			4 (15.4); 4 (+)			4 (15.4); 1 (–), 3 (+)		
		Available human assistance	4 (15.4); 1 (–), 3 (+)			2 (7.7); 2 (+)			0 (0)		
	**Hedonic motivation**			*24 (92.3)*			*13 (50.0)*			*9 (34.6)*	
		Usage pleasure	19 (73.1); 2 (–), 16 (+), 1 (±)			9 (34.6); 1 (–), 8 (+)			6 (23.1); 3 (–), 3 (+)		
		Usage interest	14 (53.8); 8 (–), 6 (+)			5 (19.2); 5 (–)			3 (11.5); 2 (–), 1 (±)		
	**Price value**			*13 (50.0)*			*10 (38.5)*			*8 (30.8)*	
		Willingness to pay	13 (50.0); 4 (–), 5 (+), 4 (±)			10 (38.5); 1 (–), 7 (+), 2 (±)			8 (30.8); 4 (–), 3 (+),1 (±)		
		Financial savings	0 (0)			1 (3.8); 1 (+)			2 (7.7); 2 (+)		
	**Habit**			*19 (73.1)*			*18 (69.2)*			*13 (50.0)*	
		Use of PA technology	15 (57.7); 6 (–), 9 (+)			15 (57.7); 6 (–), 9 (+)			12 (46.2); 6 (–), 4 (+), 2 (±)		
		Use of similar technology	10 (38.5); 4 (–), 5 (+), 1 (±)			8 (30.8); 2 (–), 5 (+), 1 (±)			3 (11.5); 2 (–), 1 (+)		
**Emerging categories**											
	**Other motivational factors**			*18 (69.2)*			*10 (38.5)*			*20 (76.9)*	
		Motivation to be related to others	14 (53.8); 4 (–), 10 (+)			9 (34.6); 7 (–), 2 (+)			19 (73.1); 4 (–) 13 (+), 2 (±)		
		Motivation for competition	6 (23.1); 6 (+)			0 (0)			1 (3.8); 1 (+)		
		Motivation for health	1 (3.8); 1 (+)			3 (11.5); 3 (+)			4 (15.4); 4 (+)		
	**Other characteristics**			*2 (7.7)*			*4 (15.4)*			*4 (15.4)*	
		Perceived reliability	0 (0)			3 (11.5); 3 (–)			1 (3.8); 1 (–)		
		Intimacy preservation	2 (7.7); 2 (+)			1 (3.8); 1 (+)			4 (15.4); 3 (–), 1(+)		
		Distraction by other technology features	0 (0)			3 (11.5); 3 (+)			0 (0)		

^a^Valence is the number of participants who expressed the different acceptability categories and subcategories negatively (–), positively (+), or neutrally (±).

^b^Categories in italics were summed based on a count of individual participants who mentioned at least one of the subcategories.

^c^PA: physical activity.

#### The Choice of Preferred Technology-Based PA Interventions and Behavioral Intentions to Use Them

Among the 26 participants, 10 indicated during the interview their preference for active video game, 9 for mobile app, and 7 for telehealth. Between the time they agreed to participate in this study and the interview, 1 participant changed her choice and preferred mobile app instead of telehealth (P17). All participants who preferred a technology-based PA intervention (ie, active video game, n=10; mobile app, n=10; telehealth, n=6) expressed a behavioral intention to use it. In addition, among the participants who preferred another technology-based PA intervention (ie, second and third choices), (1) 3 expressed low behavioral intention for active video game (ie, P8, P10, and P25), (2) 1 expressed high behavioral intention for active video game (ie, P22), (3) 1 expressed low behavioral intention for mobile app (ie, P20), (4) 3 expressed low behavioral intention for telehealth (ie, P16, P17, and P22), and (5) 4 expressed high behavioral intention for telehealth (ie, P7, P12, P14, and P26). The following excerpts illustrate these results:

(mobile app) I'll use it...well after...yeah, I think, all the time

P5

(telehealth) ah, but if I have it at home, I’ll do it all the time

P19

I can't, I can't say to myself, well I'm going to turn on a video game to do some sports

P8

but what is certain is that I’m not interested in telehealth

P22

#### UTAUT2 Constructs

##### Performance Expectancy

This category of the UTAUT2 referred to the degree to which the participants believed that the technology would be useful to them in doing PA. Three subcategories emerged for the 3 technology-based PA interventions: (1) adequacy of PA, (2) engagement and sustainability of PA, and (3) PA management support. Among the participants who mentioned the adequacy of PA for active video game (n=8), most perceived it to be of low adequacy; for example, “there is no real contact, or the descriptions are badly done, or something like that” [P2].

For mobile app (n=8), perceptions were quite good about the adequacy of PA. For telehealth, adequacy was perceived as high among the 12 participants who mentioned this subcategory; for example,

to see if we’re doing the right things, if we’re doing the exercise correctly, so that we’re not doing anything and everything

P2

Perceptions of the technologies to engage and sustain PA throughout a session or over the long term were generally positive for active video game (n=16), mobile app (n=12), and telehealth (n=14), as highlighted by the following quotes:

but maybe to start, you know, as a first step to get back into sports, it’s maybe more interesting to start with the video game

P26

(telehealth) even if it’s on the computer, it motivates us, it pushes us a little bit to improve, to go a little further

P24

For active video game (n=3), mobile app (n=13), and telehealth (n=4), participants perceived these technologies as mostly helping them to manage and monitor their PA, as noted by one of the participants: “that we can see our progress on the application.” [P6]

##### Effort Expectancy

This category of the UTAUT2 referred to the degree to which the participants believed that the technology would be easy to use for PA. First, participants mentioned the perceived ease of use in relation to the physical effort involved in PA. For active video game, participants (n=6) perceived this to a mixed degree as illustrated by the following quotes:

if, for example, he asks me to jump, I’ll jump, but uh, my knee will hurt

P4

precisely when it’s a video game, there are several levels.

P16

For mobile app (n=4) and telehealth (n=2), the effort involved in PA was perceived to be low and adapted to their capacities; for example, mobile app was perceived as “adapted to each level, so it’s good for making progress” [P7]. Second, participants perceived the effort required by the technology as low (ie, active video game, n=8; mobile app, n=14; telehealth, n=4), which refers to the concept of the usability of the technology-based PA interventions. One participant stated as follows: “(telehealth) one click and it starts up by itself, it seems very simple to me” [P8].

##### Social Influence

This category of the UTAUT2 referred to the degree to which the participants perceived that significant others believed they should use the technology-based PA intervention to do PA. Two subcategories emerged for the interventions: (1) others’ perceptions of the technology (ie, subjective norms), and (2) others’ uses of the technology (ie, descriptive norms). For all the technology-based PA interventions, others’ perceptions of the technology (ie, active video games, n=8; mobile apps, n=10; telehealth, n=4) and others’ uses of it (ie, active video games, n=6; mobile apps, n=3; telehealth, n=1) were mostly perceived positively. For example, participants stated:

everyone plays these games at least a little bit so they would find it normal [P13] (telehealth) perhaps there would be some curious ‘ah, but how does it work? Can I try to do a session with you?’

P20

(mobile app) maybe they would even use it, who knows

P6

##### Facilitating Conditions

This category of the UTAUT2 referred to the participants’ perceptions of the resources and support available to them while using the technology-based PA interventions. Four subcategories emerged: (1) anytime and anywhere usage, (2) available material resources, (3) technological knowledge, and (4) available human assistance. Participants perceived mobile app (n=22) to be usable anytime and anywhere, as noted by P23: “an application you can do it whenever you want, so when you have some time,” whereas the perception of active video game (n=17) was more nuanced: “having to be at home to do it, it’s more restrictive” [P11]. For telehealth (n=18), participants mostly perceived it as usable to a limited extent: “having to keep a schedule could be complicated for me” [P1], except for those who preferred this technology-based intervention and perceived it as adapted to their lifestyle and allowing them to save transport time: “we’re going to be able to organize ourselves more easily according to, well, our daily lives, we’re not going to lose time in transportation” [P8].

For mobile app (n=11) and telehealth (n=9), the participants felt they had material resources available, as illustrated by the following quote:

(telehealth) I've got the smartphone on which I've got a webcam, I've got the computer with it so, um well, hardware-wise I'll have everything

P8

For active video game (n=11), the necessary equipment was not always available; for example, “video games you have to have the equipment, so sometimes you can’t have it” [P23].

Technological knowledge needed to use the technology-based PA interventions was cited to a lesser extent (ie, active video game, n=3; mobile app, n=4; telehealth, n=4), but mostly perceived positively, as this excerpt shows:

(telehealth) none because, although I’m not much of a TV person or anything, I know how to use computers, plug in, connect or whatever

P20

Available human assistance for using technology-based PA interventions was reported positively for mobile app (n=2) and active video game (n=4), as illustrated by P1, “(active video game) by giving me time to do it, maybe do it with me,” but was not reported for telehealth.

##### Hedonic Motivation

This category of the UTAUT2 referred to the fun or pleasure of using technology-based PA interventions. First, participants perceived different degrees of pleasure associated with the use of the interventions. For active video game (n=19) and mobile app (n=9), usage was mostly perceived as pleasant, as illustrated by P4:

we say video game, so it's a game, so since there’s the word ‘game’ in it, we'll have more fun, we'll laugh.

For telehealth (n=6), the pleasure of using this technology was more nuanced, as the following extracts show:

telehealth will annoy me so I put it in last position

P3

with telehealth, with a coach who will be there ‘you have to do this, you have to do that’

P4

Or on the contrary:

it can also be fun

P7

telehealth, I think it’s interesting

P26

Second, participants also mentioned their general interest in the use of technology-based PA interventions. This interest in the 3 interventions was generally low for those participants who mentioned this subcategory (ie, active video game, n=14; mobile app, n=5; telehealth, n=3), except for those participants who preferred active video game and expressed high interest. For example, the participants said:

(mobile app) always have the phone for everything, choosing your groceries, looking at the bank account, now for sports...that’s a lot of phone

P1

I actually lose interest very quickly in applications in general, I think it’ll be the same

P18

(active video game) if you decide to do an hour of sports every day, [...] well, that’s still spending time in front of a screen

P2

I really like anything interactive, I know it won't have anything to do with interactive video games but I like it anyway

P12

##### Price Value

This category of the UTAUT2 referred to the participants’ cognitive trade-offs between the perceived benefits of the technology-based PA interventions for doing PA and the estimated monetary cost of using them. Participants expressed a degree of willingness to pay to use the technology-based PA interventions, provided the price was not too high. Mobile app was considered to have an acceptable price by those who mentioned this subcategory (n=10), while opinions were more mixed for active video game (n=13) and telehealth (n=8). The following quotes illustrate this:

pay for the application, I wouldn’t mind to a certain extent

P2

(mobile app) if it's in a gym or if it's my phone, um...in the gym I say to myself, if I like it I'll go, I'll pay, so it would be the same

P17

(active video game) we are not going to say that it’s within our reach

P14

(telehealth) I know that, even if it would have to be paid for, and I know that I would be willing to pay the price

P10

To a lesser extent, 3 participants also mentioned the financial savings with the technology-based PA interventions, especially mobile app (n=1) or telehealth (n=2), compared with the gym, as illustrated by P10: “I’m sure by telehealth and all of that, it would be much cheaper.”

##### Habit

This category of the UTAUT2 was extended from the original definition and referred to the previous use of the technology-based PA interventions or a similar technology. Thus, 2 subcategories emerged: (1) the use for PA of the technology described in the presentations or similar technology, and (2) the use of a similar technology for activities other than PA. For active video game and mobile app, participants described having rather a high use of similar technologies for PA (ie, active video game, n=15; mobile app, n=15) and for other activities (ie, active video game, n=10; mobile app, n=8), as illustrated by these quotes:

(WiiFit) about a week, let’s say 3 to 4 times a week

P15

I had an application, for example, for weight

P11

By contrast, for telehealth, the use of similar technologies for PA (n=12) or other activities (n=3) was rather perceived as low, as cited by P14 “in telehealth, since I’ve never tested it, so I don’t know.”

#### Emerging Categories

##### Other Motivational Factors

This category corresponded to the motivational determinants of technology-based PA intervention use that went beyond the motivational factors included in the hedonic motivation and performance expectancy constructs of the UTAUT2. Three other motivational factors emerged: (1) motivation to be related to others, (2) motivation for competition, and (3) motivation for health. The motivation to be related to others (ie, need for relatedness) referred to the motivation to use the technology-based PA interventions to be included in a group or to be connected with other people to do PA or with a coach. For active video game (n=14) and telehealth (n=19), participants perceived these technology-based PA interventions as a response to their need for relatedness:

(active video game) then I think that yeah, with an evening with friends or with children, it can be really nice.

P20

(telehealth) the good thing is, if I remember, there was the possibility to be with a coach or with a group.

P23

By contrast, mobile app (n=9) was mostly perceived as foreign to this need; for example,

the application I put it last because um being alone to do my sport is not very motivating.

P13

Motivation for competition (ie, performance achievement goals) referred to the use of the technology-based PA interventions to measure oneself against others and compare oneself in a kind of competition. This subcategory was mainly mentioned for active video game (n=6); for example, P24 considered active video games as allowing “a little competition with people.” One participant (ie, P26) also mentioned this for telehealth. Motivation for health referred to the use of the interventions to improve physical capacities, lose weight, or avoid obesity relapse. This subcategory was cited positively for active video game (n=1), mobile app (n=3), and telehealth (n=4), as illustrated in the following quote: “(active video game) I think it can give me more...endurance, cardio” [P26].

##### Other Characteristics

This category corresponded to constructs that were not included in the UTAUT2 and were not related to motivational factors. Perceived reliability was sometimes perceived as low for mobile app (n=3) and telehealth (n=1), as noted by P7: “(mobile app) if it’s stuff that grinds uh, or bugs, well that’s annoying.”

Active video game (n=2) and mobile app (n=1) were perceived by some participants as preserving their intimacy because these technology-based PA interventions did not require them to expose themselves. By contrast, 3 participants perceived telehealth as exposing them; for example,

for the telehealth, uh...negative point is that sometimes, we don’t really want to show ourselves

P26

One participant, however, considered that she was less exposed than in a gym:

and uh to do it at home without anyone around who can judge me like in a room or uh...look at me

P24

Three participants mentioned that they might be distracted by other features on their smartphone instead of using the application for PA, as illustrated in the following excerpt:

when I pick up the smartphone, well immediately my games take over, I do something else, I go to answer the phone and then I make a phone call and finally I don’t do what I went to do on my phone

P1

Beyond the study objectives, 2 participants perceived the proposed technology-based PA interventions as complementary (ie, the 3 interventions for P26 and mobile app and telehealth for P5).

## Discussion

### Principal Findings

The aim of this study was to examine the suitability of the UTAUT2 model for technology-based PA interventions in the context of bariatric surgery. To this end, we explored the reasons for preference for 1 of 3 interventions (ie, telehealth, active video game, and mobile app) to gain an in-depth insight into the factors contributing to behavioral intention to use the technology. Of the 26 participants, 10 chose active video game as their preferred technology-based PA intervention and 11 expressed a high behavior intention to use it, 10 preferred the mobile app and 10 intended to use it, and 6 chose telehealth and 10 intended to use it.

For active video game, the main positive factors mentioned by the participants were usage pleasure, engagement and sustainability of PA, and motivation to be related to others. By contrast, usage anytime/anywhere and usage interest were perceived more negatively. These specificities can serve as benchmarks for the development of future active video games targeting women in postbariatric surgery. For example, we recommend that the developers of these games stimulate usage pleasure, which could be achieved with less demanding physical exercises. For mobile app, the possibility to use it anytime and anywhere, the availability of material resources, and support for PA management were the most positively mentioned factors, while usage interest and motivation to be related to others were perceived less positively. According to these specificities, we could recommend short PA sessions or those based on everyday movements with little or no equipment. For telehealth, the adequacy of PA, engagement and sustainability of PA, and the motivation to be related to others were widely perceived positively, while telehealth was perceived as constraining for anytime and anywhere usage. We recommend that qualified professionals teach PA through this type of technology, with some flexibility in booking slots and choice of extended hours. To the best of our knowledge, this study is the first to identify the most salient factors explaining the preferences of vulnerable people regarding technology-based PA interventions.

All the UTAUT2 constructs were broken down into subcategories specifically adapted to technology-based PA interventions in bariatric surgery, differing from other technologies used in chronic diseases. For example, facilitating conditions in diabetes mobile health (mHealth) self-management are broken down into technical support, support from the mHealth app itself, and health care professionals [24]. In our study, facilitating conditions in the technology-based PA interventions were broken down into anytime and anywhere usage, available material resources, technological knowledge, and available human assistance. Although some studies have used the UTAUT2 for technology-based PA interventions [35,36], to our knowledge this is the first study to characterize in-depth the concepts of the UTAUT2 model in this context for a vulnerable population. These findings validated the suitability of the UTAUT2 model in this context. However, future studies would be necessary to extend these results to other clinical contexts.

Our results showed that factors other than the constructs of the UTAUT2 model also emerged to characterize the acceptability of technology-based PA interventions. The UTAUT2 model combines several theories, such as the hierarchical model of intrinsic and extrinsic motivation [37]. The concept of performance expectancy integrates extrinsic motivation, and the concept of hedonic motivation integrates intrinsic motivation [38]. The UTAUT2 is a recognized theoretical framework for technology acceptability [20,21], which has been extended to several contexts [25]. However, few studies have considered the specificities of the acceptability of technology-based PA interventions in light of more contemporary sociocognitive models of motivation. Our results have been discussed in relation to the Self-Determination Theory (SDT) [39] and achievement goal theory [40]. In particular, motivation to be related to others corresponds to the need for relatedness, which is one of the basic psychological needs of the SDT. The extrinsic motivation, as cited by the participants (ie, motivation for health), referred to identified regulation among the 4 types of regulation of extrinsic motivation of the SDT. These results are in line with the findings of recent studies that have examined the relations between these theories and acceptability theories [41-45].

The examination of the relationships between the concepts of motivation to PA and the constructs of the UTAUT2 model in the context of technology-based PA interventions seems to be an emerging area of research that should be encouraged. As motivation toward PA has a higher degree of generality than motivation toward technology-based PA interventions (ie, performance expectancy and hedonic motivation), the SDT constructs could be positioned as antecedents of the UTAUT2 variables.

### Limitations

Despite the several strengths of this study, some limitations must be acknowledged. The first limitation is related to the study design. The descriptions of the technology-based PA interventions provided general information and were relatively similar to avoid any bias to the presentation itself. As the descriptions were hypothetical, we cannot apply these results directly to similar real technology-based PA interventions available on the market. Although we conducted our qualitative analyses according to research guidelines [26,30-32] and reached theoretical saturation, the generalizability of our results may be questioned. First, our population was composed only of young women who underwent bariatric surgery. We can assume that young adults are rather familiar with technology. Second, those who agreed to participate in the interviews may have been more interested in technology-based PA interventions than the rest of the population. Third, there was no process for having the participants validate the results, such as member checking. Some of their responses may thus have been slightly overinterpreted.

Another type of limitation was related to our theoretical approach. The interviews were conducted within the framework of the UTAUT2 model, which means that the model constructs did not emerge naturally (ie, their frequency of citation is probably overestimated), unlike the other constructs, such as the motivational constructs. The relative weight of each of the factors in explaining behavioral intention to use technology-based PA interventions will have to be established in future studies, as will the relation with usage behavior, which was not measured in this study.

Face-to-face contact was minimized to lower the risk of virus transmission during the COVID-19 pandemic, which meant that telehealth was used extensively. As the interviews were conducted before the pandemic, perceptions about telehealth may have changed (eg, [46]).

### Conclusions

The results showed that the UTAUT2 model is suitable for examining the acceptability of technology-based PA interventions in the context of bariatric surgery. All UTAUT2 constructs were broken down into subcategories specifically tailored to this context. The results also highlighted the most salient factors explaining the preferences of vulnerable individuals regarding several types of technology-based PA interventions. These results have important implications as they could be used as benchmarks for future technology development. Although the UTAUT2 model is an integrative model, other factors of acceptability were identified. Future studies must be conducted to better examine the causal relationship between the SDT and UTAUT2 constructs.

## Appendix

Multimedia Appendix 1 Original French and English translation of the written descriptions of three technology-based physical activity interventions with illustrations.

Multimedia Appendix 2 English translation of the interview guide.

Multimedia Appendix 3 Participants’ perceptions of the different acceptability categories and subcategories.

## Abbreviations

mHealth: mobile health
PA: physical activity
SDT: Self-Determination Theory
UTAUT2: Unified Theory of Acceptance and Use of Technology 2
