# Supplementary material for: The Acceptability of Technology-Based Physical Activity Interventions in Postbariatric Surgery Women: Insights From Qualitative Analysis Using the Unified Theory of Acceptance and Use of Technology 2 Model
Source: JMIR Hum Factors. 2023 Jan 23;10:e42178. doi: 10.2196/42178 (PMC9947814; doi:10.2196/42178)

**Supplementary materials - Appendix 1**

**Original French and English translation of the written descriptions of three technology-based physical activity interventions with illustrations**

**Activité Physique Adaptée réalisée en visioconférence (Original French version)**

*Contexte :*

Pour votre santé et vous aider dans la gestion de votre poids, il est essentiel de pratiquer régulièrement une activité physique. Afin de vous accompagner au mieux dans cette démarche, nous vous proposons en complément de votre prise en charge actuelle, de participer à un programme d’activité physique basé sur l’utilisation des nouvelles technologies.

*Utilisation :*

Ce programme sera réalisé en visioconférence. Vous serez seul chez vous, mais en interaction avec les autres participants et accompagné par un professionnel de l’activité physique. Le professionnel indiquera à l’ensemble du groupe, en direct par visioconférence, les exercices à réaliser qui seront définis en fonction des capacités physiques du groupe. Pour vérifier la bonne réalisation des exercices, le professionnel de l’activité physique pourra corriger votre posture en direct, et vous pourrez échanger directement avec lui et les autres participants. Les activités physiques qui vous seront proposées seront réalisées chez vous en intérieur, à un horaire fixe préalablement défini. Différentes activités physiques vous seront proposées afin de développer vos fonctions cardio-respiratoires, musculaires, de souplesse et d’équilibre (exemples : gymnastique, yoga, stretching, renforcement musculaire…).

*Matériel :*

Ce programme nécessitera l’utilisation de votre ordinateur muni d’une webcam ou de votre smartphone. Aucun achat de matériel ne vous sera demandé. Si vous n’en avez pas, une webcam vous sera fournie, et un support technique sera mis à votre disposition.

**Telehealth (English translation)**

*Context:*

For your health and to help you manage your weight, it is essential to practice physical activity regularly. In order to accompany you in this process, we suggest that you participate in a physical activity program based on the use of new technologies, in addition to your current treatment.

*Use:*

This program will be carried out by videoconference. You will be alone at home but interacting with the other participants and accompanied by a physical activity professional. The professional will indicate to the whole group, live by videoconference, the exercises to be done, which will be defined according to the physical capacities of the group. To verify that the exercises have been carried out correctly, the physical activity professional will be able to correct your posture live, and you will be able to discuss directly with him and the other participants. The physical activities that will be proposed to you will be carried out at home, at a fixed time previously defined. Several physical activities will be proposed to develop your cardiorespiratory, muscular, flexibility and balance functions (examples: gymnastics, yoga, stretching, muscle strengthening...).

*Equipment:*

This program will require the use of your computer with a webcam or your smartphone. You will not be required to purchase any equipment. If you don't have one, a webcam will be provided to you, and technical support will be made available to you.


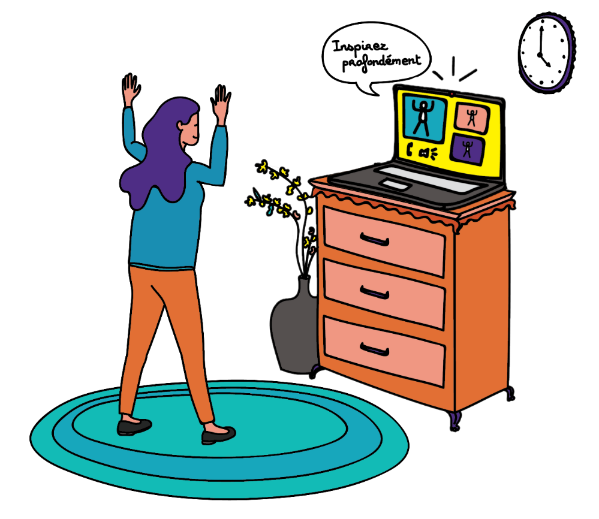


**Jeu vidéo actif (Original French version)**

*Contexte :*

Pour votre santé et vous aider dans la gestion de votre poids, il est essentiel de pratiquer régulièrement une activité physique. Afin de vous accompagner au mieux dans cette démarche, nous vous proposons en complément de votre prise en charge actuelle, de participer à un programme d’activité physique basé sur l’utilisation des nouvelles technologies.

*Utilisation :*

Ce programme sera réalisé au travers d’un jeu vidéo actif. Vous serez seul chez vous, et vous devrez accomplir des missions nécessitant la réalisation d’exercices physiques afin de progresser dans le jeu. Plusieurs niveaux de difficulté vous seront proposés afin de s’adapter à vos capacités physiques. La réalisation correcte des exercices vous permettra d’avancer dans le jeu au même rythme que votre progression physique. Vous pourrez également affronter d’autres joueurs sur les différents défis qui vous seront proposés. Vous pourrez jouer en autonomie depuis chez vous en intérieur, aux horaires qui vous conviennent le mieux. Au travers du jeu vidéo, différentes activités physiques vous seront proposées afin de développer vos fonctions cardio-respiratoires, musculaires, de souplesse et d’équilibre.

*Matériel :*

Ce programme nécessitera l’utilisation de votre télévision ou de votre ordinateur, et d’une console de jeux. Aucun achat de matériel ne vous sera demandé. Si vous n’en avez pas, une console de jeux vous sera fournie, et un support technique sera mis à votre disposition.

**Active video game (English translation)**

*Context:*

For your health and to help you manage your weight, it is essential to practice physical activity regularly. In order to accompany you in this process, we suggest that you participate in a physical activity program based on the use of new technologies, in addition to your current treatment.

*Use:*

This program will be carried out through an active video game. You will be alone at home, and you will have to accomplish missions requiring the performance of physical exercises in order to progress in the game. Several levels of difficulty will be proposed in order to adapt to your physical capacities. Correct completion of the exercises will allow you to progress in the game at the same pace as your physical progress. You will also be able to compete against other players in the different challenges that will be proposed to you. You will be able to play independently from your home, indoors, at the times that suit you best. Through the video game, several physical activities will be proposed to develop your cardiorespiratory, muscular, flexibility and balance functions (examples: gymnastics, yoga, stretching, muscle strengthening...).

*Equipment:*

This program will require the use of your television or computer and a game console. You will not be required to purchase any equipment. If you do not have one, a game console will be provided and technical support will be made available to you.


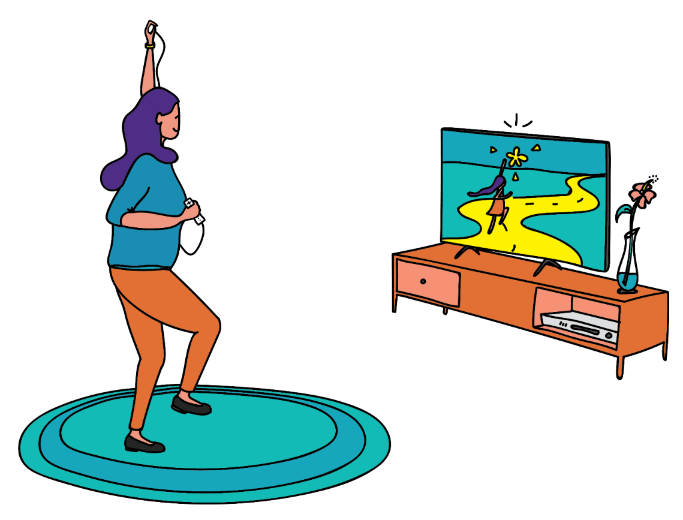


**Application mobile (Original French version)**

*Contexte :*

Pour votre santé et vous aider dans la gestion de votre poids, il est essentiel de pratiquer régulièrement une activité physique. Afin de vous accompagner au mieux dans cette démarche, nous vous proposons en complément de votre prise en charge actuelle, de participer à un programme d’activité physique basé sur l’utilisation des nouvelles technologies.

*Utilisation :*

Ce programme sera réalisé à l’aide d’une application mobile associée à une montre connectée. Vous aurez à disposition une application mobile vous incitant à la réalisation de différents exercices physiques et votre activité physique globale sera mesurée grâce à cette montre. Des défis avec plusieurs niveaux de difficulté vous seront proposés afin de s’adapter à vos capacités physiques. Vous pourrez programmer à votre convenance vos rappels d’activité. Afin de visualiser vos progrès, un rapport de votre activité physique sera accessible chaque jour sur votre application mobile, mesuré à l’aide des mouvements enregistrés par votre montre connectée. Votre activité physique sera réalisée en autonomie, en intérieur ou extérieur, dans le lieu de votre choix et aux horaires qui vous conviennent le mieux. Au travers de votre application mobile, différentes activités physiques vous seront proposées afin de développer vos fonctions cardio-respiratoires, musculaires, de souplesse et d’équilibre (exemples : gymnastique, yoga, stretching, renforcement musculaire, marche…).

*Matériel :*

Ce programme nécessitera l’utilisation de votre smartphone et d’une montre connectée. Aucun achat de matériel ne vous sera demandé. Si vous n’en avez pas, une montre connectée vous sera fournie, et un support technique sera mis à votre disposition.

**Mobile application (English translation)**

*Context:*

For your health and to help you manage your weight, it is essential to practice regular physical activity. In order to accompany you in this process, we suggest that you to participate in a physical activity program based on the use of new technologies, in addition to your current treatment.

*Use:*

This program will be carried out using a mobile application linked to a platform for managing your health data. You will have at your disposal a mobile application encouraging you to perform different physical exercises and your overall physical activity will be measured thanks to the sensors of your phone. Challenges with several levels of difficulty will be proposed in order to adapt to your physical capacities. You will be able to program your activity reminders at your convenience. In order to visualize your progress, a report of your physical activity will be available every day on your mobile application, measured with the help of recorded movements. Your physical activity will be performed independently, indoors or outdoors, in the place of your choice and at the times that suit you best. Through your mobile application, several physical activities will be proposed to develop your cardiorespiratory, muscular, flexibility and balance functions (examples: gymnastics, yoga, stretching, muscle strengthening, walking...).

*Equipment:*

This program will require the use of your smartphone. You will not be required to purchase any equipment. Technical support will be provided.


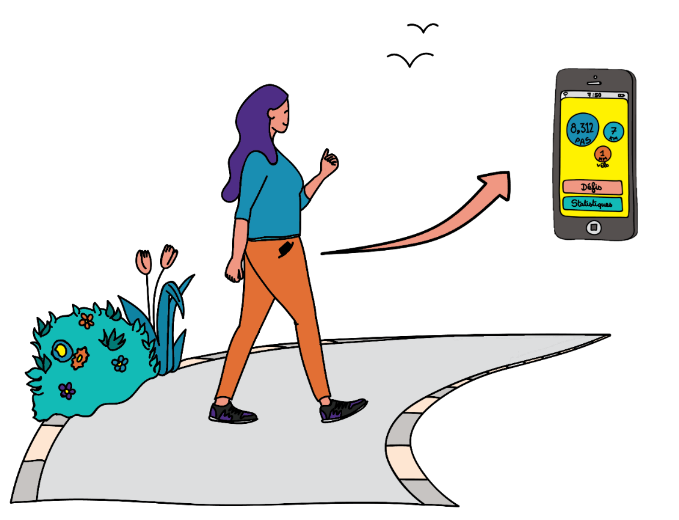

Supplement: Multimedia Appendix 1 [file humanfactors_v10i1e42178_app1.docx]
