# Supplementary material for: The Acceptability of Technology-Based Physical Activity Interventions in Postbariatric Surgery Women: Insights From Qualitative Analysis Using the Unified Theory of Acceptance and Use of Technology 2 Model
Source: JMIR Hum Factors. 2023 Jan 23;10:e42178. doi: 10.2196/42178 (PMC9947814; doi:10.2196/42178)
Supplement: Multimedia Appendix 2 [file humanfactors_v10i1e42178_app2.docx]

**Supplementary materials - Appendix 2**

**English translation of the interview guide**

**Introduction**

You have just answered all the questions of the survey on physical activity and new technologies. If you don't mind, let's go back to the last part with the different technology scenarios proposed to help you be more physically active. We will discuss your choices and preferences.

We will discuss what you like/dislike about these physical activity programs with new technologies. I would like to understand how you perceive them, how you represent them to yourself.

You are always free to refuse. If you don't mind, I will record our discussion. Only I will have access to this recording and it will be anonymized in the transcript.

**Building trust and confidence**

Before continuing, do you have any questions about what you have just completed?

Would you like to have a glass of water before continuing?

Can you tell me about your physical activity? Do you have or do you practice any physical activity? Do you have any physical activity goals for the future?

**Part One: Presentation of technology-based physical activity interventions (TbPAI) descriptions and confirmation of the ranking**

In this first part, we will try to understand your ranking of the programs for health by asking you to describe the positive and negative points for each program.

*Recall the 3 health programs, then recall the order*

You have been presented with three physical activity programs based on the use of technologies. Next, we asked you to rank them in the order of your preference - this order is *stated order*.

**Part Two: Exploration of the reasons for the ranking**

Can you explain to me your ranking of the three programs?

*Obtain information about the perception of each program*

**TbPAI 1:**

Can you explain to me what you like about this program?

Is there anything you dislike about this program?

**TbPAI 2:**

Can you explain to me what you like about this program?

Can you explain to me what made you put this program in second position?

Can you explain to me what you dislike about this program?

**TbPAI 3:**

Can you explain to me what you like about this program?

Can you explain to me what made you put this program in third position?

Can you explain to me what you dislike about this program?

*Summary of responses* - Do you have anything else to add?

**Part Three: Application of the UTAUT2 dimensions to the preferred technology, and in comparison to the other TbPAI**

Now in a third part, we will go deeper into your perceptions of your favorite program in relation to different elements and in comparison to the other TbPAI.

**Performance expectancy**

What can this program do for you?

How do you find this program useful?

What are the gains and benefits of using this program?

**Effort expectancy**

What difficulties do you see in using this program?

How much effort will using this program require of you?

How easy does using the program seem to be?

**Social influence**

How would your family, friends, and significant others perceive this program?

What is the perception of important people regarding the use of the system?

**Facilitating conditions**

Do you have the resources that would allow you to follow this program?

*(By resources, we mean your environment, materials, a space...)*

What organizational and technical structures would enable you to use it?

**Hedonic motivation**

For you, what is interesting, fun, enjoyable about these programs?

*Fun and pleasure in use*

**Price value**

You were asked about these physical activity programs on the assumption that the necessary materials would be provided. If there was a charge for this equipment, would it make a difference to you?

*Question the relationship between price and perceived benefits*

**Habits**

Did you know about this type of program to promote physical activity? Have you ever used any?

What similar programs have you used before?

Do you already use similar programs in other activities? How often?

*Ask about program usage patterns*

**Behavioral intention**

Imagine yourself, tomorrow, participating in this physical activity program:

Would you intend to use this technology?

How often would you use it?

*Summary of Responses* - "Do you have anything else to add? "

Thank you for answering my questions.
