# Supplementary material for: The Acceptability of Technology-Based Physical Activity Interventions in Postbariatric Surgery Women: Insights From Qualitative Analysis Using the Unified Theory of Acceptance and Use of Technology 2 Model
Source: JMIR Hum Factors. 2023 Jan 23;10:e42178. doi: 10.2196/42178 (PMC9947814; doi:10.2196/42178)
Supplement: Multimedia Appendix 3 [file humanfactors_v10i1e42178_app3.docx]

**Supplementary materials - Appendix 3**

**Participants’ perceptions of the different acceptability categories and subcategories**

| **Categories** | **Sub-categories** | **Active Video Game** | **Mobile Application** | **Telehealth** |
| --- | --- | --- | --- | --- |
| **UTAUT2 constructs** | |  |  |  |
| **Performance expectancy** | |  |  |  |
|  | Adequacy of physical activity | P2- P4+ P6- P7- P13- P17- P21+/- P26+/- | P2- P6+ P7+/-* P9+/- P10+ P13- P17+ P23+ | P1+ P2+ P7+ P8+ P10+ P13+ P19+ P20+ P23+ P24+ P25+ P26+ |
|  | TbPAI to engage and sustain physical activity | P1+/- P2- P3+ P4+ P7+ P9+ P12+ P13+ P14+ P15+ P16+ P18+ P19+ P21+ P24+ P26+ | P2+ P5+ P7+ P8- P11+/-* P16+ P17+ P21+ P22+ P23+ P24+ P25- | P2+* P3+ P4-* P7+ P8+ P10+ P11+ P13+ P19+ P20+ P23+ P24+ P25+ P26+ |
|  | Physical activity management support | P3+ P11- P17+ | P1+ P4+/- P6+ P7+/- P9+ P10+ P11+ P16+ P17+ P21+/- P23+ P25+* P26+ | P6+ P10+ P17+ P25+ |
| **Effort expectancy** | |  |  |  |
|  | Effort required by physical activity | P1- P2- P4- P7+ P14+/- P16+ | P7+ P17+ P22+/- P23+ | P19+ P26+ |
|  | Effort required by the technology | P3+ P4- P11- P14+ P15+ P16+ P23 +/- P26+/-* | P2- P3+ P5+ P6+ P7- P8+ P9+ P11+ P14- P16- P21+* P22- P23+ P26+ | P8+ P24+ P25+ P26+ |
| **Social influence** | |  |  |  |
|  | Others’ perceptions on the TbPAI | P1+ P3+ P12+/- P13+ P14- P15+/- P18+ P26+ | P2+* P5+ P6+ P7+/- P9+/- P11+ P17+ P21+ P22+ P23+ | P8+/- P20+ P24+/- P25+ |
|  | Others’ uses of the TbPAI | P8+ P10- P13+ P14+ P16+/- P17+ | P2+ P6+ P10+ | P10+ |
| **Facilitating conditions** | |  |  |  |
|  | Anytime and anywhere usage | P1+ P3+ P4- P6- P7+ P11- P13+ P14- P16+/- P17- P18+ P19+ P21- P22+/- P23+/- P24- P26+/- | P1- P2- P3+ P5+ P6+ P7+ P8+ P10+ P11+ P12+ P13+ P15+/- P17+ P18+ P19+ P20+ P21+ P22+ P23+ P24+ P25+/-* P26+ | P1- P3- P5- P6- P7+/-* P8+ P10+ P11- P13- P16- P17- P18- P20+ P22- P23- P24+/- P25+ P26+ |
|  | Available material resources | P2- P3+ P4+/- P12+ P13+/- P15+ P16+ P18+/-* P23+/-* P25- P26+ | P2+/- P5+ P7+ P11+ P12+ P16+ P17+ P19+ P21+ P22+ P24+ | P1+ P2+ P8+ P10+ P19- P20- P24+ P25+/-* P26- |
|  | Technological knowledge | P15+ P20- P26+ | P2+ P6+ P9+ P21+ | P8+ P20+ P24+ P26- |
|  | Available human assistance | P1+ P12-* P16+ P19+ | P7+ P17+ |  |
| **Hedonic motivation** | |  |  |  |
|  | Usage pleasure | P1+ P4+ P6+ P7+/- P8- P9+ P12+ P13+ P14+ P15+ P16+ P17- P18+ P20+ P21+ P23+ P24+ P25+ P26+ | P3+ P6+ P10+ P11+ P15+ P17+ P20- P21+ P23+ | P3- P4- P7+ P8+ P21- P26+ |
|  | Usage interest | P2- P3+ P4+ P5- P6+ P7- P8- P10- P11- P12+ P15+ P16+ P18- P20- | P1- P8- P18- P20- P25- | P2+/-* P18- P20- |
| **Price value** | |  |  |  |
|  | Willingness to pay | P1- P2+ P3+/-* P4+/-* P12- P13+ P14+/-* P15+ P16+ P18+* P23-/+* P25- P26- | P2+* P6+* P7+* P9+ P11+/-* P17+ P21+ P22+* P23+/- * P25- | P2+ P8- P10+ P19- P20- P23+/- P24+ P25- |
|  | Financial savings |  | P23+ | P8+ P10+ |
| **Habit** | |  |  |  |
|  | Use of PA technology | P1+ P3+ P4- P8- P9- P12- P13+ P14+ P15+ P16+ P17+* P18+ P19- P24+ P26- | P2+* P5- P6+ P7+ P8- P9+ P10- P14- P16+ P17+ P19- P21- P22+ P23+ P25+ | P4- P8+/- P9- P10+ P12- P14- P16+ P17- P19+ P20+/-* P24- P25+ |
|  | Use of similar technology | P2+ P3+ P4+ P5- P11- P12+ P16+ P17- P20- P26+/-* | P5+ P6+ P7+ P11+ P16- P18- P21+ P26+/-* | P20- P24+ P26- |
| **Behavioral intention** | | P1+ P3+ P4+ P8- P10- P12+ P13+ P14+ P15+ P16+ P18+ P22+* P25- P26+ | P2+ P5+ P6+P7+ P9+ P11+ P17+ P20- P21+* P22+ P23+ | P7+ P8+ P10+ P12+ P14+ P16- P17- P19+ P20+ P22- P24+ P25+ P26+ |
| **Emerging categories** | |  |  |  |
| **Other motivational factors** | |  |  |  |
|  | Motivation to be related to others | P1+ P2- P3+ P4-P6+ P7- P12+ P13+ P14+ P15+ P16- P20+ P21+ P23+ | P2- P5+ P7- P9- P10- P11- P13- P15+ P20- | P2+ P4- P5+ P7-/+ P8+ P9+ P10+ P11+ P12+ P13+P15+ P16- P17+/- P19+ P20+ P21- P22- P23+ P25+ |
|  | Motivation for competition | P3+ P6+ P9+ P19+ P24+ P26+ |  | P26+ |
|  | Motivation for health | P26+ | P5+ P7+ P17+ | P19+ P20+ P25+ P26+ |
| **Other characteristics** | |  |  |  |
|  | Perceived reliability |  | P5- P7- P17- | P14- |
|  | Intimacy preservation | P24+ P26+ | P2+ | P2- P4- P24+ P26- |
|  | Distraction by other technology features |  | P1+ P21+ P26+ |  |

Notes. * Resolved disagreements.
